# Logistics, effectiveness, safety, and accessibility: Factors determining obesity medication patient preferences from a photovoice analysis

**DOI:** 10.1016/j.obpill.2025.100238

**Published:** 2025-11-27

**Authors:** Alvin Mondoh, Francisca Contreras, Hilary Craig, Michael Crotty, Carel W. le Roux

**Affiliations:** a Diabetes Complications Research Centre, Conway Institute of Biomedical and Biomolecular Research, University College Dublin, Dublin 4, Ireland; b My Best Weight Clinic, Dublin, Ireland

**Keywords:** Obesity medication, Pharmacotherapy, Patient preferences, Type 2 diabetes, Obesity, Safety, Weight loss

## Abstract

**Background:**

Obesity is a chronic, relapsing, and multifactorial disease that necessitates sustained, patient-centred management. Although pharmacotherapy is now an integral component of obesity care, there is limited evidence regarding the factors influencing patients’ choices among specific medications. As part of the Innovative Medicines Initiative 2 (IMI2) programme. Stratification of Obesity Phenotypes to Optimize Future Obesity Therapy (SOPHIA) and the second phase of a three-part qualitative doctoral programme, the present study is built upon previous interview findings that examined how patients conceptualize and interpret the factors shaping their pharmacotherapy preferences.

**Methods:**

A qualitative Photovoice methodology was employed with treatment naive adults attending a specialist weight management clinic. Participants captured photographs reflecting factors shaping their medication choices and they also engaged in facilitated reflective interviews. Data was analysed using the MAXQDA 2024 software and the Braun and Clarke's framework for reflexive thematic analysis.

**Results:**

The photovoice analysis revealed 4 themes around patient preferences for obesity medications including a) Logistics around Lifestyle, b) Effectiveness, c) Safety, risk and tolerability and d) Accessibility.

**Conclusion:**

Patient preferences for obesity pharmacotherapy arise from the interaction of efficacy expectations with logistical challenged, while concerns regarding safety and accessibility also contribute to decision making. Clinicians should include explanations how the medications can fit into the lifestyle of patients, while also addressing structural barriers that may affect access to treatment, but ultimately allowing patients to understand the balance between efficacy and safety will allow optimal shared decision-making to support sustainable pharmacotherapy pathways.

## Graphical abstract

**Figure:**
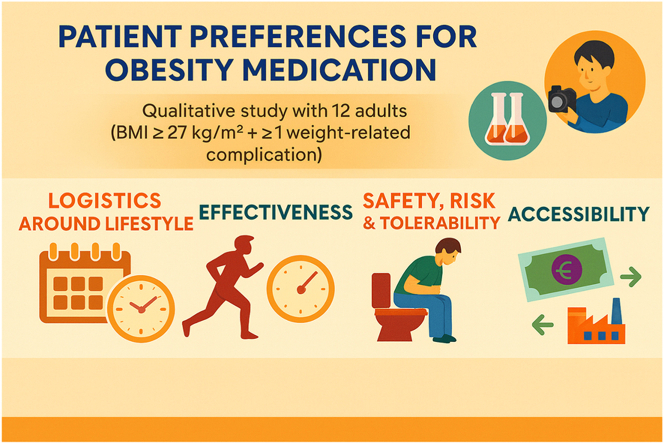

## 1 Introduction

Obesity is a chronic, progressive, and relapsing disease marked by dysregulated appetite, impaired metabolic signalling, and excess adiposity which contribute to various cardiometabolic, reproductive, and psychosocial complications [1]. The global increase in obesity poses significant challenges for healthcare systems and highlights the need for effective, patient-centred long-term management. Recent advances in obesity pharmacotherapy particularly Glucagon-like peptide-1 (GLP-1) and Glucose-dependent insulinotropic polypeptide/GLP-1 receptor agonists (GIP/GLP-1RAs) agonists have reshaped clinical treatment pathways [1]. Pharmacotherapy now plays a growing role in evidence-based obesity treatment, with modern agents showing clinically meaningful weight loss and health improvements [2]. However, real world use of these medications remains inconsistent, as many individuals discontinue treatment due to concerns about tolerability, safety, accessibility, or uncertainty regarding long term outcomes [3,4]. These challenges underscore the importance of understanding the factors that influence patient preferences for obesity medication. By identifying the determinants of patient decision making, healthcare providers can better tailor treatment plans to address patient concerns, improve adherence, and ultimately enhance treatment outcomes.

Understanding the factors that influence patient choices in obesity treatment, including preferences for specific medications is therefore essential. Previous qualitative research has demonstrated that treatment decisions are shaped not only by clinical characteristics but also by patients lived experiences, personal goals, values, practical considerations and perceptions of stigma or identity [5,6]. These findings highlight the importance of examining treatment choices from multiple perspectives to fully capture the complex reasoning underlying patient decision-making in obesity management.

This research is part of the Innovative Medicines Initiative 2 (IMI2) programme Stratification of Obesity Phenotypes to Optimize Future Obesity Therapy (SOPHIA), an international effort to advance patient-centred obesity care. It also forms one phase of a three-part doctoral research series exploring patient preferences for obesity treatment through complementary qualitative methods. The phases include individual interviews, photovoice methodology (the focus of this study), and focus group discussions. Together, using these three different methodologies will enable data triangulation and provide unifying themes and a layered understanding of how patients make treatment decisions. In the first phase, qualitative analysis of individual interviews identified five major themes: effectiveness of medication, information needed for decision-making, medication safety, practicality and individual strategies and community support in obesity management [7]. To build on these insights, the current study used Photovoice, a visual participatory research method that allowed patients to express experiences and preferences through images and narratives reflecting their daily lives [8,9]. This approach added depth to the interview-based findings from the first phase and contributed to a more comprehensive, patient-centred perspective on decision-making in obesity pharmacotherapy.

## 2 Methods

### 2.1 Study design

This qualitative study employed Photovoice, a collaborative and community-based participatory action research method that uses patient images to facilitate understanding of patient lived experiences, common issues, contexts and solutions through visual representation [8,10]. Photovoice has proven effective in obesity research, as demonstrated by studies exploring social determinants contributing to obesity [9], obesity-related stigma, and how it impacts the physical and psychosocial wellbeing of people with obesity [11].

A qualitative research design using reflective thematic analysis to explore patient preferences for obesity medication was used. This enabled the capture of the depth and complexity of individuals' experiences and preferences, particularly in relation to their personal health and decision-making processes. Thematic analysis was conducted both inductively (allowed themes to emerge from the data itself) and deductively (guided by pre-existing theoretical frameworks, such as the Health Belief Model) [12].

### 2.2 Participant recruitment

Twelve participants with obesity who expressed an interest in obesity medication were recruited. The study used purposive sampling, a non-probabilistic sampling technique that involved selecting participants based on specific inclusion criteria that aligned with the research objectives. Ethical approval was obtained from University College Dublin (Reference number: LS-23-74-LeRoux). Patients provided written informed consent and data collection occurred between June and August 2024.

The inclusion criteria for participants were: Age between 18 and 70 years; Body mass index (BMI) ≥27 kg/m^2^ with at least one weight-related complication (e.g., type 2 diabetes, hypertension, or dyslipidemia) and treatment-naive to obesity medication, meaning they had not previously taken medications specifically prescribed for obesity, but were interested to start obesity medication. Data on participant ethnicity was not collected because the relative ethnic homogeneity of the study population in Ireland offered limited analytical value for the qualitative thematic analysis. Given the small sample size, collecting such data could compromise guarantees of anonymity and participant confidentiality. Participants were excluded if they had severe psychiatric disorders, were planning or had undergone bariatric surgery, or were currently pregnant or breastfeeding. Participation in the study was entirely voluntary, and no incentives or payments were provided to participants for their time.

### 2.3 Data collection

Participants were first introduced to the purpose of the photography activity, which was to visually capture images that explained their preferences for obesity medication. The images were also able to explain the factors influencing the patient's decision-making regarding different pharmacotherapy options. They were informed that the photographs would serve as a means to express their priorities, concerns, and perspectives in ways that might not be fully conveyed through verbal communication alone.

To ensure a shared baseline of knowledge, participants viewed a 10-min informational video outlining pharmacotherapy options currently available in Europe, as well as those in late-stage development. The medications discussed included orlistat, naltrexone/bupropion, liraglutide, semaglutide, tirzepatide, cagrisema, survodutide, and retatrutide. For each medication, the video described the mechanism of action, potential benefits supported by phase two or three randomised controlled trials, anticipated weight-loss outcomes, and common side effects. This standardised presentation enabled participants to make informed reflections when taking their photographs.

Following the video, participants were encouraged to capture images symbolizing which medication or medications they believed would best suit them, and the reasons for these preferences. Participants were instructed not to photograph themselves, other individuals, or any identifiable people, in order to maintain privacy. Each participant was provided with a disposable camera, given two weeks to complete the task, and supplied with a prepaid, self-addressed envelope for returning the camera. Once the photographs were developed, they were used as visual prompts in a follow-up Zoom interview lasting 30–60 min. During the interview, participants were invited to explain the meaning of their images and how these related to their medication preferences. The interviews were primarily open-ended to allow for participant-led discussion, of which key prompts were used, such as: “Which medication option do you feel best suits you and why?” and “Which photograph best represents this choice?”.

### 2.4 Data analysis

The 12 transcripts were anonymized and were input in the MAXQDA 2024 software by two independent coders for analysis using the Braun and Clarke's six-phase thematic analysis framework [13] which included: (i) familiarization of the data, (ii) generating initial codes, (iii) searching for themes, (iv) reviewing themes, (v) defining and naming themes and (vi) producing the report. Data was interpreted to explore and understand the determinants of patient preferences in obesity medication as well as the factors that guided patient preferences for obesity medication. To enhance analytic rigor, a second researcher independently coded all interview transcripts. The two coding frameworks were compared, and discrepancies were resolved through team discussion until agreement was reached on a harmonized set of themes and sub-themes, thereby increasing the validity and credibility of the findings.

## 3 Results

The purpose of this study was to examine the themes that drive patients’ decision making between different obesity medication. The four main themes identified included: a) Logistics around Lifestyle, b) Effectiveness, c) Safety, risk and tolerability and d) Accessibility as in Table 2.

### 3.1 Patient demographics

The study included 12 participants with BMI of ≥27 kg/m^2^, all of whom were female, with ages ranging from 18 to 75 years as summarized in Table 1. The most prevalent health condition was obesity, affecting all participants. Other common co-morbidities included pre diabetes, dyslipidemia, gastroesophageal reflux disease, hypertension and gallbladder disease and infertility.

### 3.2 Theme 1-logistics around lifestyle

Patients consistently evaluated medications through the lens of their daily routines, work patterns, and personal commitments. Dosing frequency, route of administration, and discretion in use were key determinants of preference.

#### 3.2.1 Dosing frequency, route administration and discretion in use

**Fig. 1:**
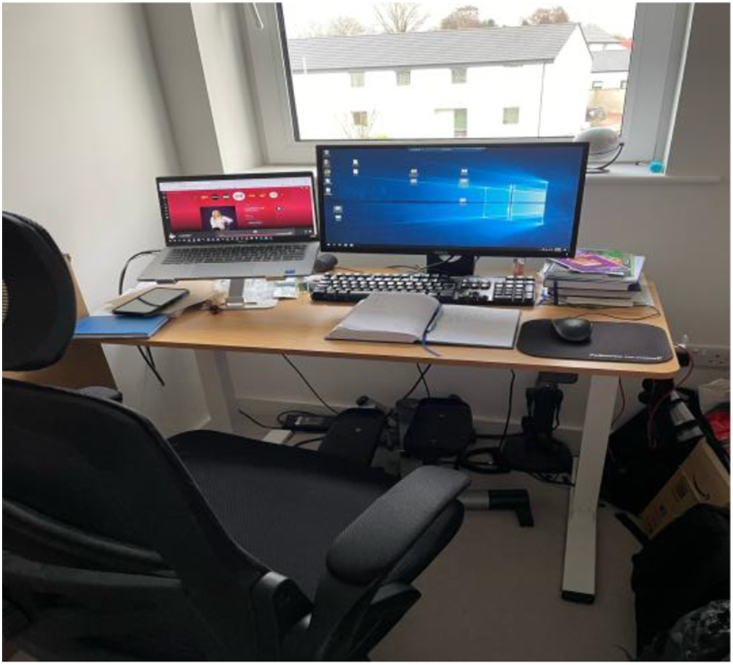
Work station.

Respondent 3 expressed a strong preference for medications that could be administered on a weekly basis rather than daily. They highlighted the practical challenges of managing daily doses, particularly when working long hours as symbolized by Figure 1. Daily medications required meticulous planning around their shifts, and they often had to carry doses to work to ensure timely administration. In contrast, weekly medications offered more flexibility, allowing them to plan their doses on weekends when they were off work.

*“I would prefer something like weekly … You have to carry all these medications to work so that you can have it on time. Sometimes it might not be on time, because then, if you're working long hours, then you have to plan according to your shifts, so if it's weekly you can. I can plan it in a way that I'm having it just like on a weekend, and then I know it will overlap on the weekends that.” (Respondent 3,*Figure 1*)*

**Fig. 2:**
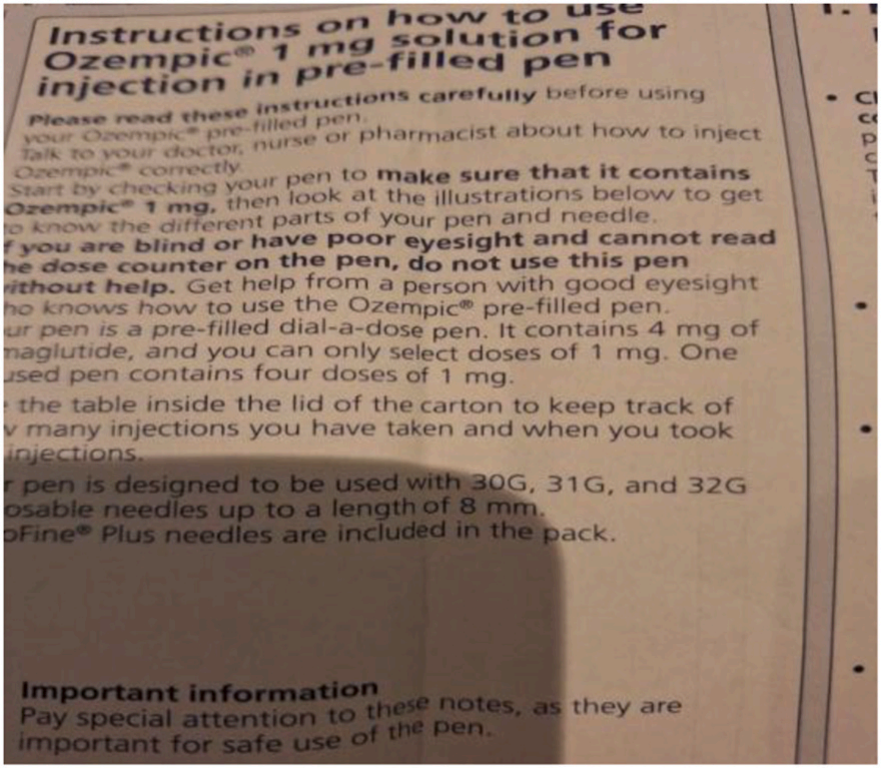
Semaglutide instructions leaflet.

Respondent 7 expressed a preference for oral medications (Figure 2), noting that oral medications were straightforward to use, requiring only knowledge of the correct dosage. They mentioned having previous experience with self-administering injections, which reduced their concern but not entirely. Despite their experience, they still felt a lingering anxiety about ensuring proper administration, as they were not trained medical professionals.

*“Oral ones would be fine. You're just taking a tablet. All you have to know is how many of them to take. I have taken injections before, so I wasn't extremely concerned, because I know I have done it on my own before, and I managed but it's still a concern … Because I'm not a trained medical professional. I am putting a medication into my body, and I want to make sure that I'm able to do it properly. (Respondent 7,*Figure 2*)*

Lifestyle integration emerged as critical for adherence. Weekly dosing, portability, and discreet administration were seen as essential attributes of a preferred treatment, shaping patients’ likelihood of initiating and continuing pharmacotherapy.

### 3.3 Theme 2-effectiveness

Efficacy expectations strongly shaped medication preference, with patients setting personal weight-loss targets and seeking agents that aligned with these aspirations. Appetite suppression and “food noise” reduction was particularly valued. Among the twelve participants, tirzepatide and semaglutide emerged as the most preferred options with tirzepatide accounting for 50 % (n = 6), followed by semaglutide at 33.3 % (n = 4), orlistat at 8.3 % (n = 1), survodutide and retatrutide at 8.3 % (n = 1). Participants expressed clear preferences for specific obesity medications, primarily tirzepatide and semaglutide, based on perceived efficacy, ease of use, and alignment with lifestyle needs. Other medications such as orlistat, survodutide, and retatrutide were mentioned less frequently, usually in the context of curiosity rather than a strong preference.

#### 3.3.1 Weight loss

**Fig. 3:**
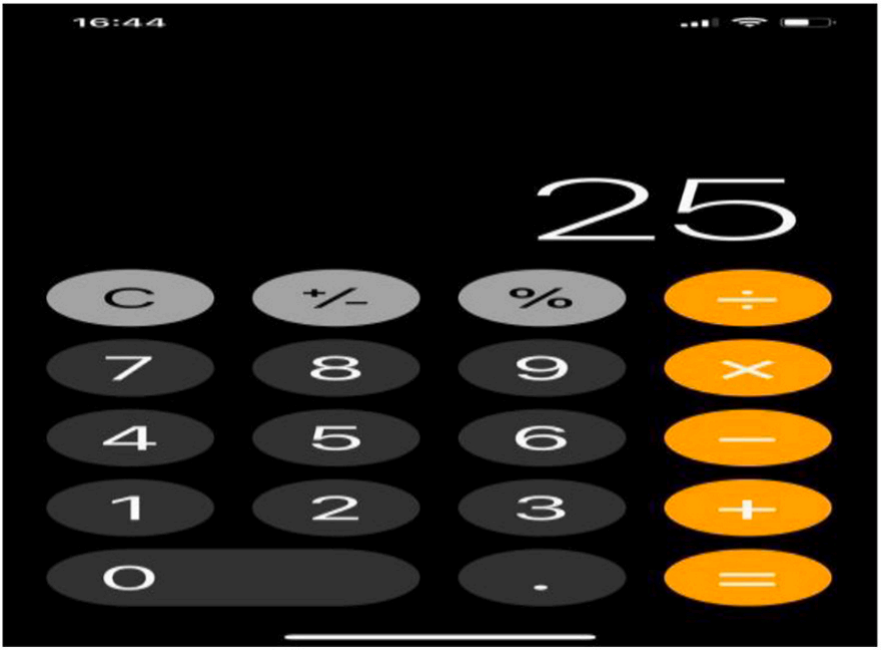
25 % weight loss of body weight.

Figure 3 of a calculator illustrated Respondent 1's targeted weight loss of 25 % reflecting their distinct health goals and medical considerations.

*“Yes. So, this one is both a goal and a barrier. So in that 25 would be the percent. So I got out the calculator when I realized that that all these drugs have an average weight loss. For, let's say, anywhere between 5% and 25% of the body weight and so I thought, Okay, how much do I actually feel like I need to lose to be within a healthy range … There are drugs that will reach those average losses but it would be barriers for other drugs for me to consider, because they wouldn't be able to reach that percentage.” (Respondent 1,*Figure 3*)*

#### 3.3.2 Appetite control

**Fig. 4:**
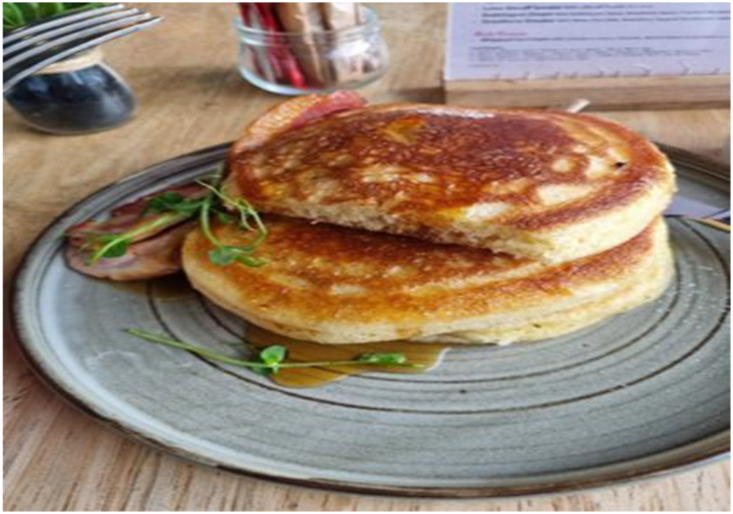
Stack of pancakes.

Participants anticipated reduction in hunger, and curbing of cravings making it easier to sustain healthier eating patterns. They expected medication to quiet “food noise,” reduce susceptibility to environmental temptations, and break habitual eating routines, thereby supporting more deliberate and balanced food choices. Respondent 5 captured the preference for semaglutide through a photograph of pancakes (Figure 4), representing their ongoing struggle with sweet cravings. They anticipated that semaglutide could promote satiety after smaller meals, reduce the frequency and intensity of cravings, and ease the mental preoccupation with food.

*“If that was a preference, I'd definitely go for it, because it's something that I'm struggling with right now, like I just can't help myself to not want to eat something sweet.” (Respondent 5,*Figure 4*)*

#### 3.3.3 Health gains: improved reproductive health

For some participants, the anticipated benefits of obesity medication extended well beyond weight reduction itself, encompassing broader physical health improvements and the possibility of reversing obesity complications. These anticipated health gains were often deeply personal and tied to long-term life goals, including enhanced mobility, reduced pain, improved reproductive health, and increased longevity. Nonetheless, their narrative reflected a deliberate, informed, and proactive stance: having undertaken detailed research, consulted with healthcare professionals, and considered the urgency of their health goals, they determined that the benefits of tirzepatide outweighed the risks.

**Fig. 5:**
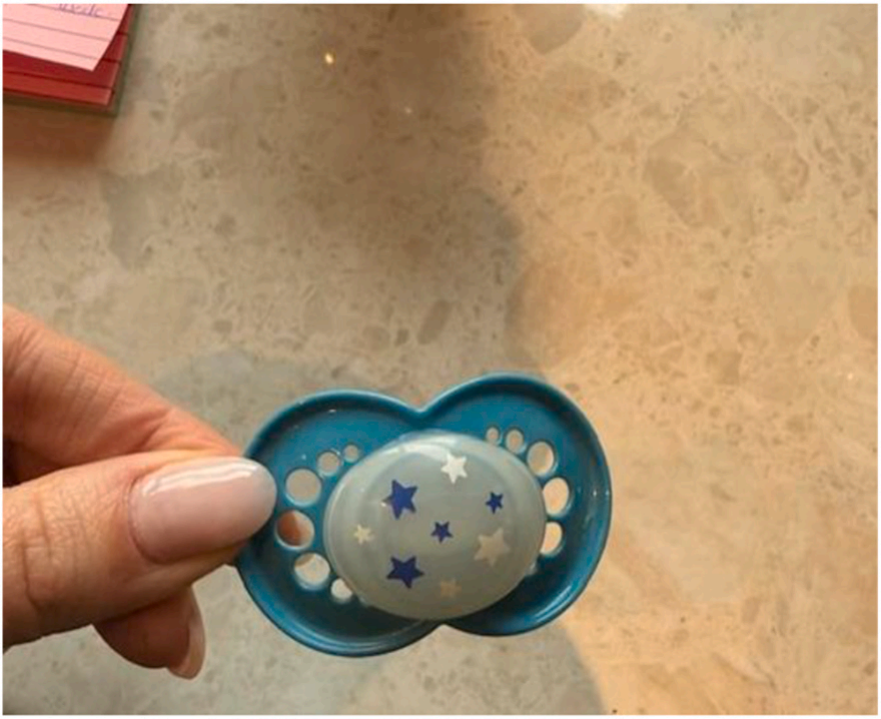
A pacifier.

This was evident in Respondent 4's choice of tirzepatide, a medication that represented a pivotal step forward which was chosen with both caution and resolve in pursuit of meaningful health improvement. The respondent also added an image of a pacifier (Figure 5) represented which presented her aspiration to become pregnant and hold a child again. The image encapsulated her ultimate goal of achieving a body capable of sustaining pregnancy and motherhood. She explained:

*“… when you’re so heavy like me, and you’ve so much things you want to achieve, that risk. I’m going to have to take it. Tirzepatide is the one that I want to try. So the pacifier as an end goal. I would love to have lost enough weight to be able to get pregnant again, hold a pregnancy and be able to physically hold a child again.” (Respondent 4,*Figure 5*)*

#### 3.3.4 Health gains: reduced cardiovascular risk and fitting into clothes

**Fig. 6:**
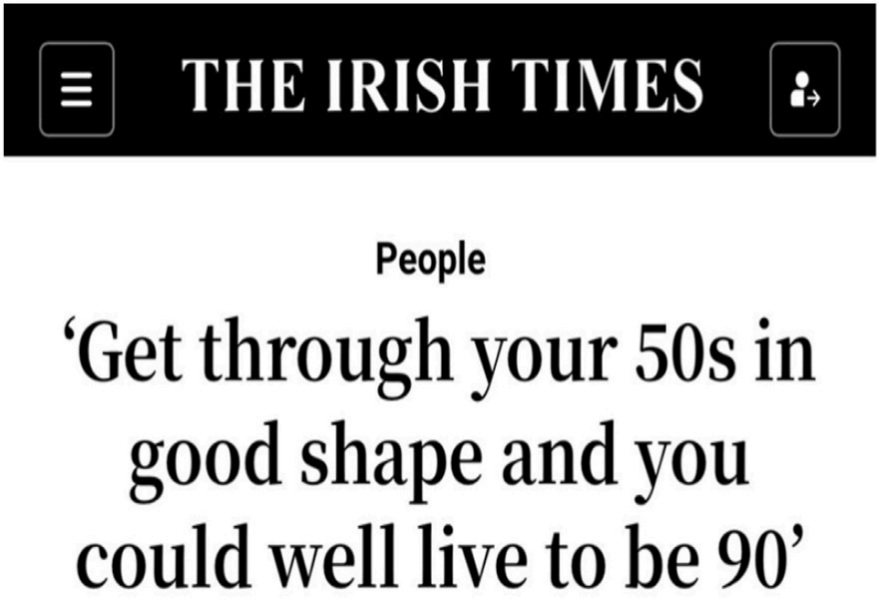
Newspaper.

According to Respondent 9, she chose tirzepatide for its proven efficacy in significant weight loss and this was driven by the personal history of the family members who died of heart attack. In reference to Figure 6 of a newspaper article, she also found a definitive goal that directly addressed her anxiety about reaching the same age at which their father passed away.

*“So, it was the tirzepatide that I decided to pick. Particularly, it was the one that was going to give the best weight. Because I've always heard that your 50s is like nice for allies, that it's the year, the decade of the most, and it's called risk in health. I had always heard that my own dad died of a heart attack and he was just turned 58, his father was 51 and died suddenly and all my uncles. So, I just thought, I'm in my 50s, mid 50s and it's coming up to the age of my dad died, so I just wanted to make sure to really do whatever I could to lose weight.” (Respondent 9,*Figure 6*)*

**Fig. 7:**
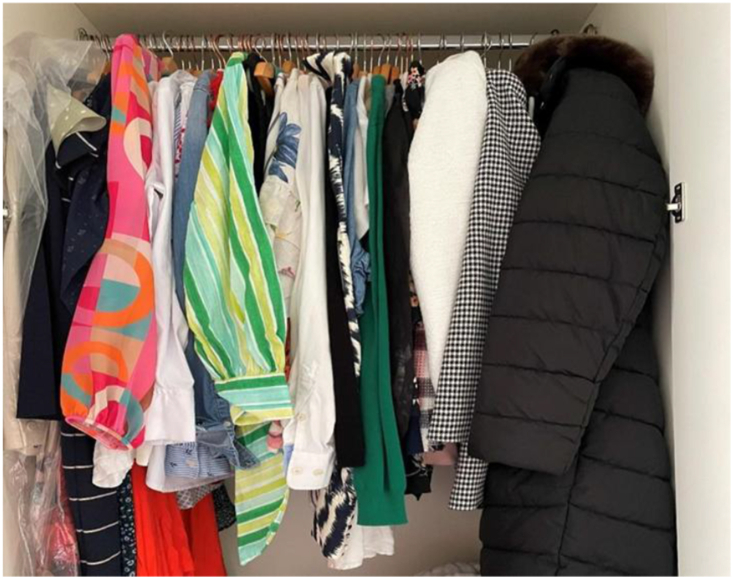
Pieces of clothing.

Since respondent 3 was also actively working towards her weight loss, her choice of using tirzepatide proved that the she was willing to use the medication as it had a higher weight loss as compared to others. She also shared a similar struggle tied to clothing and body image. She expressed a desire to fit back into beloved pieces of clothing they had outgrown (Figure 7), some of which were purchased as motivational items. Their ultimate goal was to feel comfortable in their body again without having to buy new clothes for their current size.

*“I'd say tirzepatide because definitely like the average weight loss for it is, you know, a bit higher than the rest. My end goal will be to finally fit back into most of these clothes. I am at a stage where I do not want to buy to fit my current self. I would love some of these, you know, many beloved pieces. Some I never even got to, you know, wear them. There's actually a couple of pieces there that I bought as motivation. So my end goal is definitely to be able to fit into them.” (Respondent 3,*Figure 7*)*

Patients’ medication preferences were partly shaped by efficacy expectations, as they expected to meet weight-loss targets, the ability to suppress appetite, or broader health and life goals such as improving reproductive health, enhancing body image and reducing risks of obesity complications.

### 3.4 Theme 3-safety, risk and tolerability

Perceptions of safety and side effects were integral to decision-making. Patients weighed the potential benefits against risks, sometimes using vivid imagery to convey their concerns.

#### 3.4.1 Approved treatments with longer track record

**Fig. 8:**
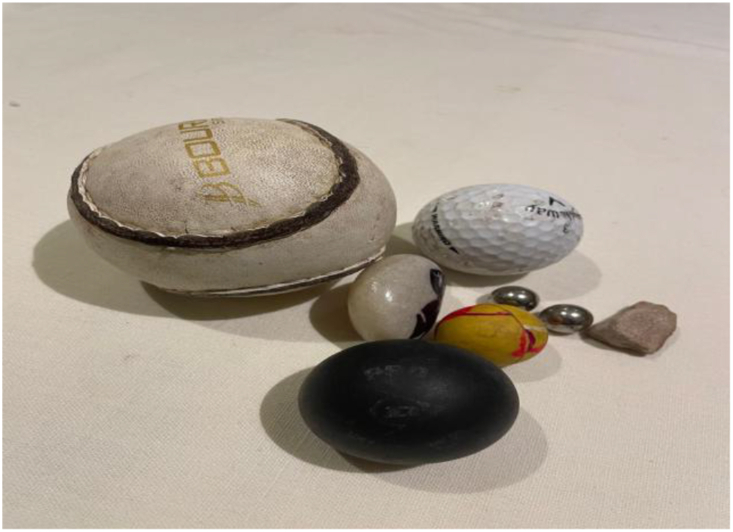
Different kind of balls.

Respondent 1 used Figure 8, to show different kinds of balls, metaphorically representing gallstones. This emphasized the physical health risks that shape Respondent 1's careful evaluation of weight-loss treatments. The image highlighted their commitment to prioritizing safety and established efficacy in their health decisions. Respondent 1 expressed concerns about the potential risks associated with unapproved drugs, specifically mentioning gallstones as a worry. They highlighted a preference for using medications that have been approved for longer over those still in clinical trials, such as survodutide and retatrutide.

*“So, these represent the concerns that I would have about getting gallstones. I know that there are two survodutide and retatrutide that are in clinical trials and I would prefer to take something that's already been fully approved for longer.” (Respondent 1,*Figure 8*)*

#### 3.4.2 Side effects

Respondent 7 expressed caution about potential side effects of certain weight loss medications, they emphasized the importance of researching possible effects, even if they might not experience them all. The respondent acknowledged that this type of medication was new to them, which prompted their thorough investigation of potential risks.

*“When I was thinking about it, and I was looking into the different medications and watching the video and like all of them. The side effects are explained of each, and I know when you take a medication you don't necessarily suffer from a side effect a lot of the time. You mightn't have any of them, but you do have to take into account the ones that are possible. So, I had to. I had to read up on them and take them into account, and.” (Respondent 7)*

**Fig. 9:**
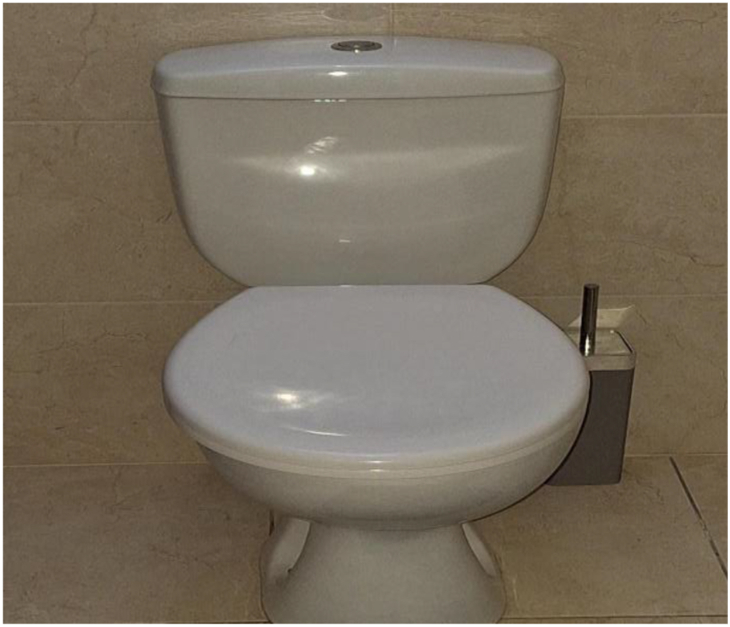
Toilet.

Moreover, Figure 9 portrayed a toilet, symbolizing Respondent 7's struggle with irritable bowel syndrome (IBS) and its impact on their daily life. The image highlighted the importance of avoiding medications that could worsen IBS symptoms, such as frequent bowel movements or nausea, which would interfere with their ability to manage their busy schedule effectively. The toilet served as a visual representation of the respondent's primary concern balancing weight-loss efforts with maintaining digestive stability and minimizing disruptions caused by IBS. This has been highlighted in the below quote.

*“I have IBS. I have a very busy life, and if I need to go to the bathroom instantly, sometimes I don't have the time or space, or the facility to do that. I was worried about feeling sick … So I needed to pick a medication that wasn't going to cause adverse reactions on my digestive system either way.” (Respondent 7,*Figure 9*)*

**Fig. 10:**
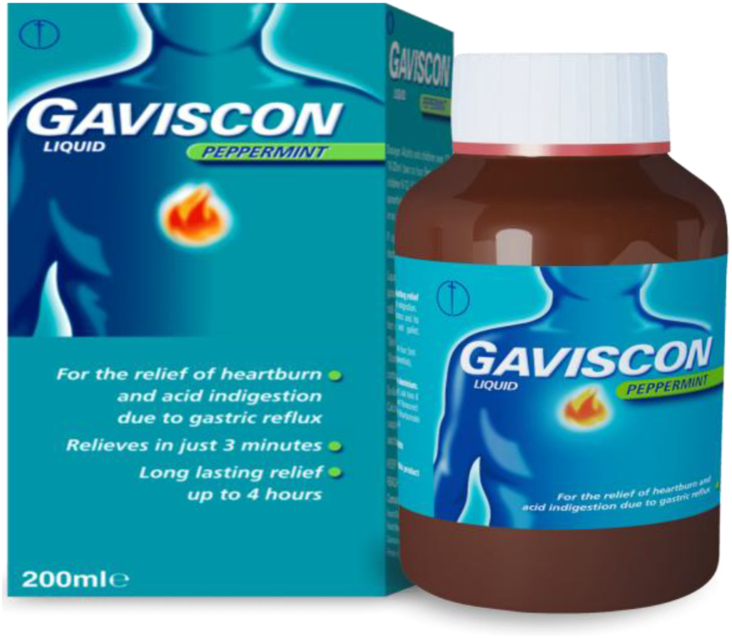
Gaviscon medication.

Respondent 2 discussed the progression of their heartburn over the years and their reluctance to take additional medications beyond what they already used for this condition (Figure 10). They expressed concern about potential side effects of certain weight-loss drugs, such as nausea or diarrhoea, which could significantly impact their quality of life and discourage them from continuing treatment.

*“So it's one of my concerns will be that I'd be … very nauseous. I know initially, probably I would be, but I just wouldn't like to be constantly sick and constantly … with diarrhoea. I think that would be a huge impact on my lifestyle” (Respondent 2,*Figure 10*)*

**Fig. 11:**
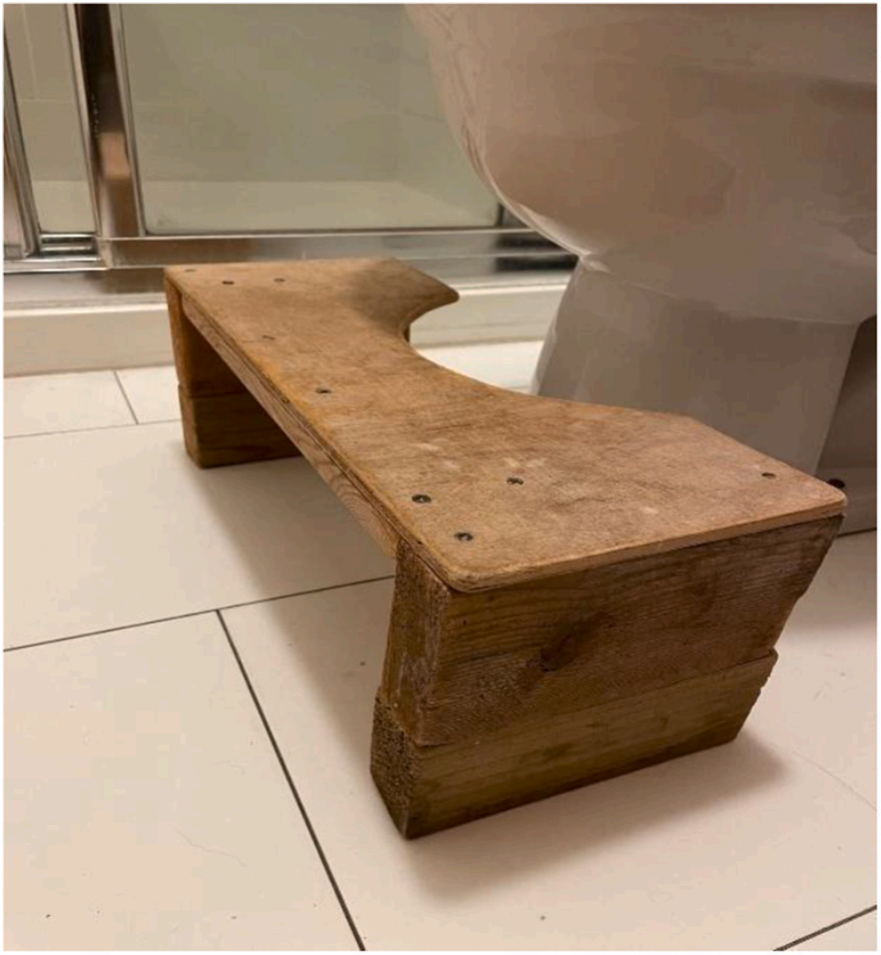
Footrest for use in the toilet.

Respondent 1 described lifelong struggles with constipation, which they feared could worsen with certain medications. They shared how this issue had become an ingrained part of their life, even requiring adaptations like using a footrest to improve posture during bowel movements. They were particularly concerned about certain medications that might aggravate this condition further, emphasizing the emotional and physical toll it had already taken on them. Figure 11 highlighted the physical and emotional toll of living with this condition and served as a visual reminder of how deeply it affected their routine and decision-making regarding medication.

*“I have struggles with constipation already and it's difficult for me to manage it. So, it would be a concern in that if I would happen to have more constipation. Then I'm going to really struggle, but if it's the opposite then I would be happy …” (Respondent 1,*Figure 11*)*

**Fig. 12:**
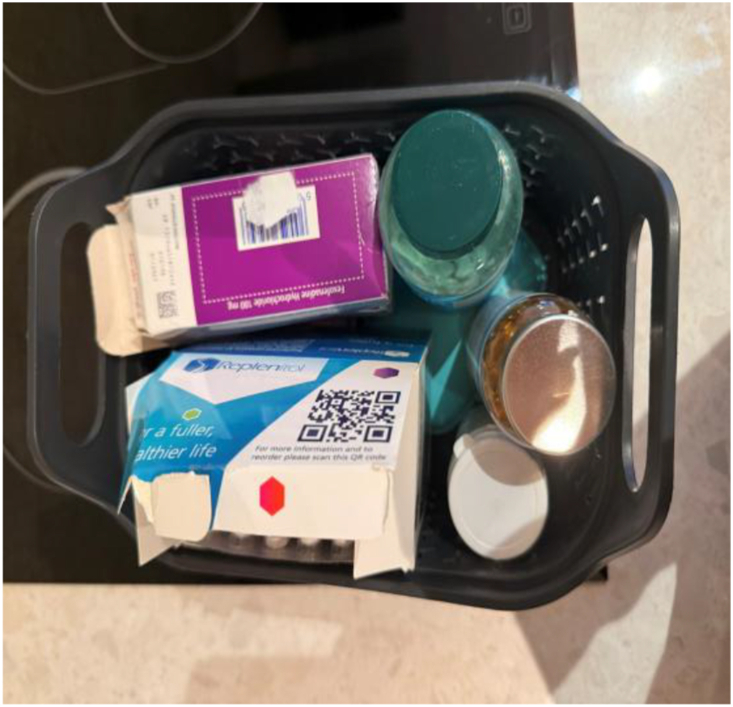
A number of medications.

Respondent 4 voiced concerns about how weight loss drugs might interact with their existing medications and health conditions with a photo of her current medications (Figure 12). They described their ongoing struggle with inflammatory arthritis and worried that weight loss drugs could exacerbate their current symptoms of fatigue and lack of energy. The respondent's apprehension stemmed from their perception that certain weight loss drugs could be “severe on the body”**.**

*“Yeah, so that medication box. So, a barrier for me, would be my current health will impact results due to tablets and all that kind of stuff I have to take. So yeah, it's just when choosing a weight loss drug, just to make sure like that that the drug wouldn't interfere with medications for me and things like that … Interactions, will weight loss be slower for me on a certain drug, because of the stuff that I'm already on …” (Respondent 4,*Figure 12*)*

### 3.5 Theme 4-accesibility

The accessibility of medications was recurring concerns. Participants evaluated long-term cost implications alongside potential reimbursement schemes and ethical considerations about access.

#### 3.5.1 Affordability

**Fig. 13:**
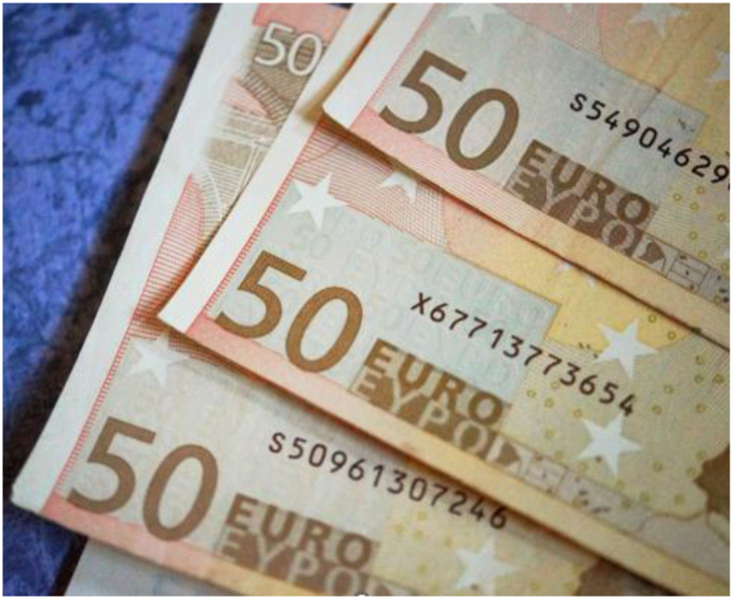
Euros cash money.

The accompanying image of the Euro money (Figure 13) visually captured the financial burden of certain medications described by the respondent. This visual element concretized the abstract worry about cost of certain drugs, making the participant's lived experience of financial strain more immediate and relatable.

*“The cost of certain medications is quite high. So being on it long term or lifetimes. And wow! It's a lot of money to invest. I know I'm investing myself, but it's just … What if the boiler goes and I need to replace the boiler? Am I sacrificing my medication, and then does that lead to not being effective, or I've taken a gap on it, or it's just it's the cost of the medication is a concern.” (Respondent 8,*Figure 13*)*

**Fig. 14:**
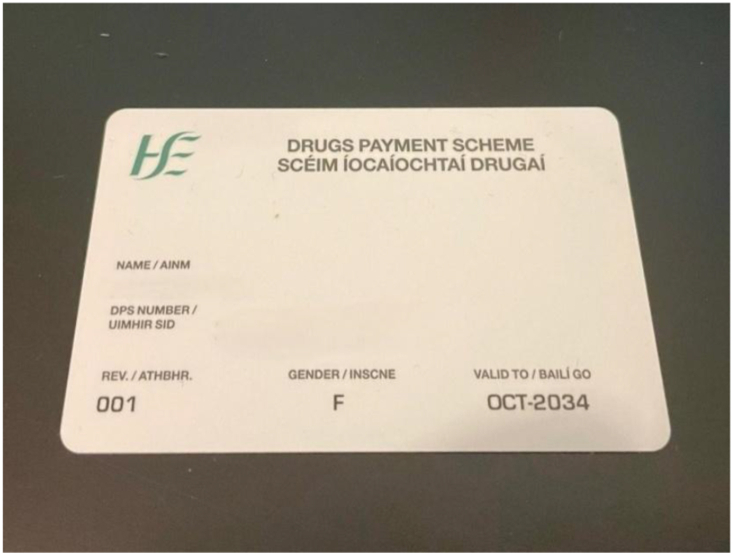
Drugs payment scheme card.

Respondent 1 highlighted cost of certain medications as a significant factor in their decision-making process, though it was not the sole determining factor. They expressed interest in understanding whether the medications were covered under a government's drug payment scheme which would impact their financial obligations. Beyond cost, they also considered other critical aspects such as potential side effects and the duration of treatment required. Figure 14 featuring a drugs payment scheme card, representing the financial aspects influencing their decisions symbolized potential cost management through payment schemes.

*“It's not necessarily a deal breaker, but it's whether … these drugs are part of the drug payment scheme here. Cost, I think, is something that requires consideration. I would probably go for the more expensive one but it would depend.” (Respondent 1,*Figure 14*)*

The above quotes highlight the complexity of cost considerations in choosing weight loss medications. While cost is a significant concern, it is part of a broader evaluation that includes health outcomes, treatment duration and financial sustainability. These reflections emphasize the need for affordable solutions that balance financial constraints with effective health management. The focus also moves from price of the medication to the perceived value proposition of taking the medication long term.

#### 3.5.2 Availability

**Fig. 15:**
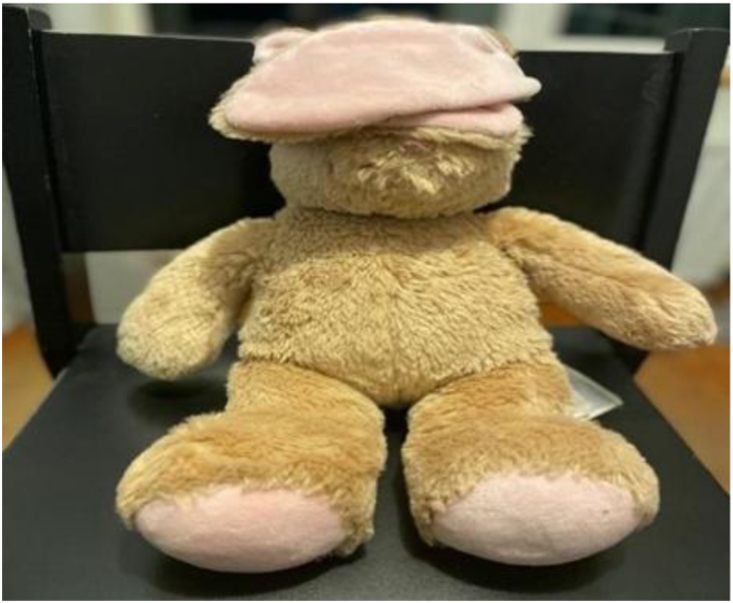
A teddy bear with one closed eye.

Respondent 1 highlighted supply issues in the past as a potential barrier to accessing obesity medications. They referred to recent shortages of certain drugs, especially those originally developed for diabetes, and expressed concerns about social shame if patients with diabetes were deprived of their medicines due to others using them for weight loss. Although they noted no current shortages, they were worried about frequent disruptions and limited availability. The teddy bear with one closed eye in Figure 15 symbolized the uncertainty and partial accessibility of weight-loss medications due to supply shortages. It reflected Respondent 1's concerns about the potential scarcity of these drugs. The image also conveyed the emotional weight of navigating ethical dilemmas, as Respondent 1 expressed fears of social shame if patients with diabetes were deprived of essential medications due to their use for weight loss.

*“Okay … the fear of social shame and that would be if any diabetics were deprived of their medicines and I'm referring to the recent news about certain drugs being like out of stock being short supply … To me that would feel shameful, it would be social shame.” (Respondent 1,*Figure 15*)*

Financial sustainability, insurance coverage, and medication supply were decisive factors. Concerns about shortages and the ethical implications of obesity medication use during supply constraints also influenced patient choices about certain drugs.

## 4 Discussion

This Photovoice study offered insights into how patients make decisions regarding obesity pharmacotherapy in real world contexts. Four primary key themes emerged: a) Logistics around Lifestyle, b) Effectiveness, c) Safety, risk and tolerability and d) Accessibility. While a previous phase of one on one interviews indicated that patients considered efficacy, safety, and practicality when selecting medications [7], the present study demonstrated that these decision-making processes are deeply embedded within daily routines, personal identity, social relationships, and systemic constraints. Consistent with broader obesity treatment literature, participants approached medication selection as an adaptive strategy to manage daily demands, psychosocial burdens, and long term health objectives rather than as a discrete decision [5,6]. The Photovoice methodology revealed dimensions of decision making rarely captured in interview only designs. Visual representations of time pressures, family responsibilities, logistical challenges, and work-related constraints highlighted how the daily realities of living with obesity shaped perceptions of acceptable and sustainable pharmacotherapy. Comparable Photovoice research has shown that visual data can expose structural determinants such as stigma, environmental barriers, and competing social obligations that patients may not express verbally [8,9].

Participants prioritized weight loss to address obesity complications and life milestones. Safety and side effects were critical considerations, particularly for respondents with pre-existing conditions like irritable bowel syndrome or heartburn while fears of medication interactions and long-term affordability also influenced decisions. Psychosocial themes emerged strongly, with body image struggles and social anxiety linked to weight and mental health challenges underscoring the emotional toll of obesity. Practical barriers, such as sedentary work environments weather related inactivity and mobility limitations further complicated adherence. Moreover, images symbolizing shame, concealment and moral tension around obesity medication were used to indicate sociocultural narratives about obesity continue to shape patient preferences. These nuanced insights advance the field's understanding of how stigma and identity influence medication decisions [14]. Respondents emphasized holistic approaches, combining medication with lifestyle changes like hydration, nutritious meals and pet-assisted exercise. These findings highlighted the need for patient-centred strategies that balance efficacy, safety, and quality of life considerations.

### 4.1 Efficacy: numbers, but beyond numbers

Several participants discussed efficacy using quantitative measures such as kilograms lost, body mass index (BMI) reductions, or percentages from clinical trials. These metrics provided a sense of certainty, comparability, and optimism. However, the quantitative data from the participants served as an initial reference, but did not capture the entirety of patient experiences. Participants also described efficacy in terms of relief from persistent thoughts about food, improved ability to manage cravings, and the possibility of living without ongoing mental negotiation regarding eating.

### 4.2 Safety, side effects, and lay risk–benefit analysis

Concerns regarding safety and tolerability, especially gastrointestinal side effects of certain medications, were prominent among participants. While patients expressed caution, they did not consider risk to be an insurmountable obstacle. Many participants evaluated discomfort in relation to personal objectives such as improved mobility, fertility, or cardiovascular health. This approach to risk-benefit analysis demonstrated a pragmatic perspective, in which patients acknowledged the trade-offs inherent in medication use and are prepared to tolerate side effects when the expected benefits are substantial.

In a randomised and qualitative study respectively, tolerability emerged as a recurrent determinant of engagement with pharmacotherapy and also the lack of comprehensive long term trials raised questions about the safety of the obesity medications [7,15]. Aligning with the above, the need for more long-term data through patient assessment, individualization of pharmacological interventions and adherence to maximise on the risk-benefit is important [16].

### 4.3 Cost, access, and structural constraints

Structural barriers such as medical cost, supply instability, and inequitable access emerged as prominent determinants of patient preference. Participants reported anxiety and concern regarding ongoing affordability, supply shortages, and ethical dilemmas related to selecting medications that may be inaccessible to others. For other patients’, price was less important than the perceived value proposition of the medications. These findings reflect broader concerns in the health policy literature regarding GLP-1 shortages, cost-related nonadherence, and socioeconomic inequities in obesity care [17].

Despite the clinical advances in the highly effective obesity medications, their high cost poses a major challenge in balancing equitable access and affordability [18]. These concerns are similar to studies with wider debates about obesity medication costs and logistics [4]. Without robust reimbursement schemes, access to newer drugs risks being stratified by income, with the affluent able to sustain treatment while others are excluded [19,20]. Considering the prevalence of the disease of obesity and its cardio-kidney-metabolic complications, the healthcare budgets are likely going to expand [21]. These findings underscore the role of structural vulnerability as a critical determinant or real world uptake, highlighting the need for health systems to address affordability, continuity of supply and insurance-related barriers. This calls for obesity to focus on long-term health and additional research regarding costs of optimal care for patients with obesity [22]. Given the large unmet need and limited resources available, adopting less effective weight-maintenance strategies can be more efficient [18]. For policymakers, this study underscores that patient preference will remain aspirational unless structural barriers to affordability and supply are addressed.

### 4.4 Implications for clinical practice

This study extended previous SOPHIA research by demonstrating that decision making regarding obesity pharmacotherapy is multidimensional, relational and shaped by broader social determinants of health. It confirmed that patient's evaluations of medications integrate obesity medications expectations with lived experience, emotional meaning, interpersonal contexts and structural constraints. The triangulation of this Photovoice study with prior interview findings and the upcoming focus group discussion data will strengthen the evidence base supporting patient-centred obesity treatment pathways.

This study demonstrated that efficacy is interpreted both through quantitative outcomes and lived experiences, with patients referencing trial data as a basis for discussing relief from cravings and enhanced social participation. Additionally, the findings show that patients actively considered risks and side effects within broader value systems. The study also identified symbolic and structural factors influencing preferences, such as identity, stigma, cost, and equity, which are not typically addressed in clinical trial reports.

The use of Photovoice was instrumental in revealing these dynamics. The images and narratives generated by patients, including depictions of everyday foods, family artefacts, and personal objects, highlighted the emotional and symbolic aspects of medication choice. These insights are challenging to obtain through quantitative methods such as use of surveys. The findings supported the value of participatory methods for exploring the emotional and contextual factors influencing patient decision-making.

Furthermore, the findings indicated that clinical discussions regarding obesity pharmacotherapy should begin by eliciting patients' definitions of success, acceptable trade-offs, and relevant constraints. Shared decision-making should explicitly include discussions of lifestyle compatibility, tolerability thresholds personal values, social circumstances and structural barriers [17]. Hence, clinicians can use specific questions to enhance these discussions, such as, “What impact would you be willing to tolerate on the logistics of your lifestyle?” or “What are the most important changes you hope to see with this treatment?” or “How do you define success beyond just weight loss?”. Additionally, asking, “What side effects are you willing to tolerate for potential benefits?” and “How does cost or access impact your decision?” can help identify patient priorities. Acknowledging patients as active participants in risk-benefit evaluation may strengthen clinician-patient partnerships and enhance adherence.

### 4.5 Strengths and limitations

A key strength of this study lies in its participatory design, after patients viewed a standard information video on all obesity medication options, which empowered patients to articulate perspectives often overlooked in clinical discourse. However, limitations included the single-centre Irish context, small sample size, and treatment-naive participants limit generalizability. Preferences were captured before treatment was started, and future longitudinal studies should examine how preferences shift as patients initiate, discontinue, or resume treatment. In addition, while this study is grounded in an Irish context, its insights could resonate with other healthcare settings, particularly those with similar resource constraints or sociocultural attitudes towards obesity. In more resource-rich environments, the emphasis on patient-centred outcomes may still hold, altering only how these insights are applied. Moreover, exploring similar participatory designs in diverse global settings could enrich understanding, highlighting both universal and unique elements across cultures. Such comparative studies could expand the frameworks of treatment efficacy and accessibility, contributing to more globally relevant obesity care strategies.

## 5 Conclusion

This study demonstrated that patient preferences for obesity medications cannot be reduced to trial outcomes. Patients may talk about efficacy in numerical terms, but even then, they can move beyond the numbers, situating efficacy in lived experiences of relief, autonomy, and belonging. Side effects are not disqualifying barriers but negotiated trade-offs. Preferences are shaped as much by logistical, and structural realities that affect access as by pharmacodynamics. To deliver treatment that is both effective and meaningful, clinicians must recognize that pharmacotherapy success depends not just on prescribing an efficacious agent but on aligning treatment with patients' daily contexts, identities, and long-term aspirations. These findings may help clinicians have better conversations with patients before prescribing obesity medications by explaining how well medications could fit into the lived, social, and structural realities of patients’ lives. Moreover, providing advice on how access can be addressed and finally framing the safety of the medication within the efficacy of the treatments may help patients make an optimally informed decision.

## Key takeaway messages

• Decisions are shaped by a balance of medication's logical fit with their lifestyle, perceived effectiveness, concerns about safety and side effects and practical issues of cost and accessibility.
• Patients are solely decision makers who perform personal risk-benefit analyses that weigh potential side effects and costs against personal health goals such as reducing cardiovascular risk, improving fertility and achieving a better quality of life.
• Clinicians and policymakers must adopt a broader, patient-centred approach. Success should be redefined using a “Whole-Life Efficacy” framework that values holistic well-being.

## Ethics statement

This human study was approved by Human Research Ethics Committee – Sciences (UCD School of Medicine) with approval number LS-23-74-LeRoux. All adult participants provided written informed consent to participate in this study. Written informed consent was obtained from the individual(s) for publication of the details of their medical case.

## Author contributions

(AM) Alvin Mondoh: Conceptualization, Methodology, Investigation, Data Analysis, Writing - Original Draft, Project Administration.

(FC) Francisca Contreras: Data Curation, Writing - Review & Editing.

(HC) Hilary Craig: Writing - Review & Editing.

(MC) Michael Crotty: Supervision, Data Analysis, Writing - Review & Editing.

(CW) Carel W. le Roux: Supervision, Conceptualization, Funding acquisition, Writing - Review & Editing.

## Declaration of AI use

The authors declare that they have not used any type of generative artificial intelligence for writing of this manuscript, nor for the creation of images, graphics, tables or their corresponding captions.

## Funding

SOPHIA has received funding from the Innovative Medicines Initiative 2 Joint Undertaking under grant agreement No. 875534. The funding source had no role in the study design, execution, data analysis, manuscript conception, planning, writing, or the decision to publish. All the authors of this paper are part of SOPHIA.

## Conflict of interest

The authors have no conflicts of interest to declare.

## Figures and Tables

**Table 1:** Participant Characteristics (n = 12)

**Age**	18–75 years
**Sex**	All Female (n = 12)
**Health Complications**	
Hypertension	2
Obesity Class I	9
Obesity Class II	3
Gastroesophageal Reflux Disease (GERD)	3
Infertility	1
Gallbladder Disease	2
Pre-diabetes	3
Dyslipidemia	3

**Table 2:** Summary of the themes and sub-themes

Themes	Sub-themes	Patient quotes
**Theme 1-Logistics around lifestyle**	**Dosing frequency, route administration and discretion in use**	*“I would prefer something like weekly cause then daily. It's kind of, you know. You have to carry all these medications to work so that you can have it on time. Sometimes it might not be on time, because then, if you're working long hours, then you have to plan according to your shifts, so if it's weekly you can. I can plan it in a way that I'm having it just like on a weekend, and then I know it will overlap on the weekends that I'm off then and in terms like of when I was looking at the video there, I see some medications that can cause nausea and vomiting and all these things.” (Respondent 3)* *“oral ones would be fine. You're just taking a tablet. All you have to know is how many of them to take. I have taken injections before, so I wasn't like I wasn't extremely concerned, because I know I have done it on my own before, and I managed but its just, it's still a concern” (Respondent 7)*
**Theme 2-Effectiveness**	**Weight loss**	*“Yes. So this one is both a goal and a barrier.So in that 25 would be the percent. So I got out the calculator when I realized that all these drugs have an average weight loss. For, let's say, anywhere between 5 % and 25 % of the body weight and so I thought, Okay, how much do I actually feel Like I, I need to lose to be within a healthy range and to me between 20 and 25 % is what I think I need to loose. So that would be my goal and there are. There are drugs that will reach those average losses but it would be barriers for other drugs for me to consider, because they wouldn't be able to reach that percentage.” (Respondent 1)* *“Like I suppose I would, in a way, but I don't want something I don't even worry about. If something has to be fast, or you know, obviously I would love to pick the one that will help you lose the most, but I do believe in as well as in slow and steady, so I wouldn't mind if it was only like a pound or 2 pound a week, or you know it doesn't have to be this instant and kind of crazy weight loss. I would like something that was like kind of going to change my life forever. Not just for the time that I'm on something, you know and like looking at long term about changing everything like. So I don't mind like. Obviously it'd be great to have one that would be quicker but then, like I am also, I have tried things where it's a quick fix, and then it just it doesn't last. So I'd rather something just slow and steady.” (Respondent 6)*
	**Appetite control**	*“So, I think, in combination with the medication, I need to make a more concerted effort to continue eating the healthy foods I do eat … I think fibre, protein, and water are the*3 most *important things … batch cooking will save time … which allows me to have 3 or 4 days of food.”(Respondent 9)* *“Progress the switch. So that's something that I will need to cut out, and that's 1 of my barriers at the moment. But I need to remove. I don't crave them, but I think the problem is, I sit down. I have a glass of wine, and it's like an automatic. I go get some crisps, and then sure, I'm yeah. The South is off, I know, is low, fast but definitely tortillas aren't sweet as the wine, and so I think I need to cut out both of them because I don't cut out the wine. Then I will eat the tortillas. I don't even really like tortillas. I actually just it's a habit. I think it's the best time with the medication. It's a new start for everything.” (Respondent 2)*
	**Health gains**	
	**Improved reproductive health**	*“I would love to be able to get back there again and keep up with everyone else, because I always felt like everyone was doing better than me there, or, you know, lifting heavier weights, and I got to the point where I was just embarrassed to go. So I just cut it out altogether then.” (Respondent 6)*
	**Improved functionality and exercise**	*“Well, if I get into a good habit of doing the 5 km at least 5 times a week as well, then I don't have to completely rely on medication. It also helps me to keep fit. It helps to keep the blood flowing, oxygenate my blood and keep me healthy.” (Respondent 9)* *“ So my treadmill or a walking pad. Actually, that's why, yeah. So, I put that as a concern, that will, I will I do enough exercise? Will I need to do a lot of exercise to achieve maximum results? Because at the moment I can't exercise much and then another side of that is, that's kind of it's a concern, and it's a goal. I would love to be able to do it properly but yeah, it's just that with my condition, or whatever it is at the moment, would a weight loss drug work as effective when I'm not exercising like the average human being.” (Respondent 4)*
	**Fitting into clothes**	*“Eem yes, my end goal will be to finally fit back into most of these clothes. Eem and obviously add into it, I think, I am at a stage where I can't, I do not want to buy to fit my current self. I would love these are some of these are, you know, many beloved pieces. Some I never even got to, you know, wear them. There's actually a couple of pieces there that I bought as motivation. So my end goal is definitely to be able to fit into them.” (Respondent 3)* *“The mirror … thank you thanks anyway. The mirror represents, I suppose me as well, I hate looking in the mirror. I feel I feel almost repulsed by myself.” (Respondent 2)*
	**Improved mental well-being**	*“Another photo you'll see. Obviously, I have anxiety. Not all to do with weight, but a large majority of it would be to do with that. As a result, you know what I mean like crowds and all that crap. Yeah. So one of my goals would be to feel better on my wedding day as a result of taking the weight loss, injection. Obviously, I know there's a lot more work to it, but that would be one of my reasons.” (Respondent 4)* *“The weighing scale is kind of represents a concern for me, I suppose. But completely associated with just sadness at the moment and wondering will I ever be able to be, stand on it again, and not be sad about what I see on it my whole life. I was very, very thin, and even when I was a child like it was, I was so thin to the point where, like I'd nearly have to go and try and see if there's anything I could do to put on weight, and so kind of the size and weight that I am now is very unhealthy, and I just I want to kind of get back to a healthy, not too thin, obviously, but just back to a healthy, manageable size. So the weighing scales I kind of. I would like to be able to associate it again with just saying that you're healthy rather than worrying every time you stand on it.” (Respondent 6)*
**Theme 3-Safety, and tolerability**	**Approved Treatments with longer track record**	*“So, these represent the concerns that I would have about getting gallstones. Okay, so this could be either a goal or a barrier, because whether or not it's approved unapproved drug, because I know that there are two survodutide and retatrutide are in trial in clinical trials and um I would prefer to take something that's already been fully approved would be my preference.” (Respondent 1)*
	**Side effects**	*“That is a tricky one. If I wasn't you'll see another barrier, that is, you know, clock time. If I was younger and there wasn't a clock against me say wedding wise pregnancy wise, I would go. I would probably pick the one that didn't have as much side effects that I'd have to be on it longer term if that makes sense.” (Respondent 4)* *“then I like it would be a lot of consideration, because obviously I'd have to look at the pros and the cons of having both you know. Do I stay overweight and have possible, you know, long term side effects down the line?” (Respondent 2)*
	**Fatigue**	*“Yes, for me. Why I've done that is because I am on other medications. At the moment. I have suffered. Obviously, I'm going into my*5th *year of suffering severely with this. Well, I've suffered for a long time, but since I had my child, and obviously the inflammatory arthritis. So why, I'm thinking even more of side effects is because I feel like I'm suffering so much at the moment. I'm really afraid that a weight loss drug would add to this kind of lack of energy, tiredness, pain, not pain but yeah, lack of energy and tiredness. Does that add to it, because I know obviously a weight loss drug is severe on the body.” (Respondent 4)*
**Theme 4-Accessability**	**Affordability**	*“Yeah, this again was around the concerns. The cost of the medication is quite high. So being on it long term or lifetimes. And wow! That's the. It's a lot of money to invest” (Respondent 8)* *“I would definitely go for the more expensive. Given that you're only going to use it for a shorter period of time.” (Respondent 3)*
	**Availability**	*“ Okay. So this one I would consider to be a barrier that might influence my decision on a particular drug type or drug name whatever because of the fear of social shame and that would be if any diabetics were deprived of their medicines and I'm referring to uh some of the if you know the recent news about certain um and I know a lot of drugs have been affected uh being like out of stock being short supply. Uh ever since the pandemic, but in particular I know a lot of the not all the drugs that you've listed, but a lot of them were originally developed for diabetics, and I wouldn't want to be one of these people, because I've heard people talk that say what there's no, there's not enough supply for diabetics and these other people are using it or taking the medicine and using it for weight loss, and that to me would. if it would feel shameful, it would be social shame. So this also relates to supply and scarcity. So I did a quick check, and I know that well, from what I can see that there are currently no market shortages. However, I would worry if something went out of supply very often, or you know that it was limited, or whatever.” (Respondent 1)*

## Abbreviations

**GLP-1**: Glucagon-Like Peptide-1
**GIP/GLP-1RAs**: Glucose-dependent insulinotropic polypeptide/GLP-1 receptor agonists
**IMI 2**: Innovative Medicines Initiative 2
**SOPHIA**: Stratification of Obesity Phenotypes to Optimize Future Obesity Therapy
**BMI**: Body Mass Index
**OM**: Obesity Medications
**IBS**: Irritable Bowel Syndrome
**GERD**: Gastroesophageal Reflux Disease
